# Notes and News

**Published:** 1888-06-09

**Authors:** 


### Notes and News.

Collected and Communicated.

Messrs. N. M. Rothschild have given a donation of £21 towards the development of the objects of The Hospitals Association.

The Brompton Consumption Hospital treated last year 13,623 new cases, and had a daily average number of 232 occupied beds. The necessary annual expenditure for the maintenance of the present measure of efficiency is more than £22,000.

The twenty-fifth annual edition of Herbert Fry's "Royal Guide to the London Charities" has just been issued by Messrs. Chatto and Windus. This well-known book of reference needs no commendation from us. Everyone who is interested in charitable work should have it. The price is eighteenpence.

The Princess Louise, Marchioness of Lorne, opened an industrial exhibition on behalf of the funds of the Recreative Evening Schools' Association. The exhibition is held at 7, Hyde Park Place, the town house of Mr. Cyril Flower. It is stated that there are nearly half-a-million boys and girls in the metropolis who have commenced to earn their own living. The Association seeks to carry on the education of these young people from the point where it now stops short. In London alone there are more than a hundred evening schools working under its auspices. The teachers and helpers are volunteers; and more than four hundred have been enrolled from among all classes of society. More volunteers are wanted in every department, and a thousand pounds is urgently required.

Although the Mary Wardell Scarlet Fever Convalescent Home at Stanmore has been open for only three and a half years, during which time it has received 680 patients, it is nearly nine years since the lady whose name is attached to it began labouring to establish it; and she is anxious that the tenth year of her work should see the building plans carried out and the debt now burdening the home paid off. At present, owing to the incomplete state of the buildings, adult male patients cannot be received, which greatly limits the usefulness of a very valuable institution. Ordinary convalescent homes, it is well known, do not receive patients recovering from infectious diseases, although these often need a change to country air before returning to their ordinary occupations more than others, and the average lodging-house keeper is shy of scarlet fever patients, and must be bribed to take them on exceptional terms. Therefore, not only the poor, but the middle class, have cause to be grateful for such an institution as the Mary Wardell Home, and it is to be hoped that its finances will soon permit of its completion.

On May 18th a shocking suicide occurred at the Victoria Park Hospital for Consumptive Patients, London. It appears that an inmate named Christopher Wheeler, aged 48, was observed by some of his fellow inmates to get out of bed and slash his throat with a table knife. An attempt was made to prevent him further injuring himself; but he slipped away and jumped from the window, alighting on the ground with a frightful crash. When picked up life was found to be extinct.

The physicians of the London Temperance Hospital, in a Supplementary Report, point out what they consider the advantages of treating patients without alcohol. They particularly desire to express the conviction that there is a wise and generous toleration of opinion in the profession on the subject of the prescription of alcohol in disease. Under these circumstances the report considers it is only necessary for the medical staff to persevere in a system of carefully observing and accurately recording results, by which alone the soundness of the principle upon which the hospital is established can be determined. Nearly 5,000 in-patients have been treated during the year, of which about half were non-abstainers. Of the whole number of cases no fewer than 704 came from the country.

The City of London Lying-in Hospital, City Road, has the good fortune to be a popular charity, and has hitherto been able to pay its debts, and retain a small balance to its credit at the bankers. Its prosperity is well deserved. Intended for the reception of poor married women, it gives them the care and skilled attendance which their circumstances would otherwise make unattainable to them during their confinements. During last year 356 women were received, and 358 children born in the hospital. Only one of the mothers died, this being the only death since May, 1886; a fact the more noteworthy and creditable, that a hospital receives a much larger proportion of difficult and dangerous cases than are met with in ordinary practice. The out-patients numbered 1,204, of whom only one woman has died; and as many of the out-patients were attended by the pupil midwives, such a low rate of mortality bears satisfactory testimony to the good training given at the British Lying-in Hospital, under the doctors and the matron, Miss Sara E. Allen. A hundred and ten nurses and twenty-four midwives were trained during the year, whose fees formed no inconsiderable part of the income of the hospital.

Outside the cover of the annual report of the Wolverhampton General Hospital, just received, is a very well-executed sketch of the hospital buildings. From the point of view of one who has to look through such reports in large numbers every week the picture is a decided advantage. It gives at a glance a general impression of the character of the hospital, and of the work which is likely to be done there. It is useful too for purposes of comparison in matters architectural. Other hospital managers would do well to take the hint here conveyed; because, if a pleasing sketch of a building disposes a hardened hospital critic to feel favourably towards the institution, the probability is that the general public, from whose pockets subscriptions and donations are to be charmed, would be similarly impressed. The hospital is large and the average daily number of in-patients for 1887 has been 118. The number of out-patients during the year reached 11,641, of which nearly five thousand were cases of accident. The total expenditure was under £7,000, and the average cost per occupied bed per annum only £45 8*s.* 4*d.*, a very modest and highly creditable sum. An amount of over £40 for dripping, &c., sold during the year, shows a careful vigilance on the part of the matron. In the items of expenditure there is no line for stimulants. The drug bill, however, is considerable, and stimulants may have been, as they properly should be, included in it.

The following vacancies are announced this week, the amount of salary in each case being given after the name of the institution :—

**Resident Medical Officers.**

Brecon Infirmary. Resident House Surgeon.—£100 per annum.
Metropolitan Asylums Board Western Fever Hospital, Fulham, S.W. Resident Clinical Assistant.—Board.
Miller Hospital and Royal West Kent Dispensary, Greenwich Road, S.E. Junior Resident Medical Officer.—£30 per annum, with board.
Nottingham General Hospital. Resident Surgical Assistant.—No salary; board, etc.
Parish of Lochs, Stornoway. Medical Officer.—£140 per annum, with house.
Royal South Hants Infirmary, Southampton. House Surgeon.—£100 per annum, with board, etc.
Sheffield General Infirmary. House Surgeon.—£120 per annum, with board, etc.
Sheffield General Infirmary. Assistant House Surgeon.—£80 per annum, with board, etc.
Surrey Dispensary, Great Dover Street, Southwark. House Surgeon.—£120 per annum.
Wellingborough and District Medical Institute. Medical Officer.—£280 per annum, and fees, with dwelling house, etc.

**Non-resident Medical Officers.**

Baltinglass Union. Medical Officer, Dunlain Dispensary.—£135 per annum, and fees.
Board of Works for the Wandsworth District. Medical Officer for the parish of Clapham.—£75 per annum, with increase.
Duffus Parochial Board. Medical Officer.—£35 per annum.
King's College Hospital. Assistant Surgeon.
London Temperance Hospital, Hampstead Road, N.W. Surgeon.
Queen's College, Birmingham. Assistant Medical Tutor.
Royal London Ophthalmic Hospital, Moorfields, E.C. Curator and Librarian.—£120 per annum.
Westport Union. Medical Officer, Westport No. 2 and Louisburgh No. 2 districts.—£39 per annum, and fees.

A church parade was made on Sunday in South London by the Sons of the Phœnix. A collection was taken on behalf of the Evelina Hospital for Sick Children.

The annual sermon and collection in aid of Ryde Infirmary was held on Sunday, May 15th, at St. Thomas's Church, when the Foresters attended in procession. Several serious accidents have lately been treated at the infirmary.

Through the kindness of Mrs. Mallock the nurses of the Torbay Hospital were able to spend a most enjoyable afternoon in the grounds of Cockington Court, and were afterwards entertained at tea on the lawn. Such acts of kindness cost nothing to those who have the power to do good, except the will and the thought. Such acts are fully appreciated by nurses, who are as a rule an intelligent set of women, and even a *little* sympathy makes their lives brighter and helps them to work more cheerfully; and, lastly, it is a good thing to shed a little brightness into the lives of others not so favourably situated.

The quarterly general court of the Governors of the Middlesex Hospital was held in the Board Room of the Hospital on Thursday, May 31st, at twelve o'clock noon, Major Ross, M.P., in the chair. There were present: The Rev. Frederick Brindly, M.A. (chaplain), Lieut.-Colonel Hollway, Andrew Clark, Esq., F.R.C.S., David W. Finlay, Esq., M.D., Walter F. Larkins, Esq., J.P., and others. The Secretary-Superintendent read the minutes of the last quarterly general court, which, after some remarks from the chairman, were confirmed. In respect of various charitable bequests, eleven executors were unanimously elected honorary governors of the hospital, and the election committee for 1888-9 duly constituted.

That part of London which lies towards the North Pole is in a condition of considerable and increasing excitement at the present time. For seven years it has been striving, not indeed with all its might, but with a respectable measure of strength, to build itself a new hospital. The hospital is at last erected, though not completed, and their Royal Highnesses the Prince and Princess of Wales have promised to perform the opening ceremony. Hence the excitement and the fluttering of fair ladies up and down the "shopping" thoroughfares in unusual numbers. The men, as becomes their superior dignity, are more stoical in demeanour, but there is every reason for concluding that in their case it is the still waters that run deep. North London is excited, and North London is pleased. The "oldest inhabitant" does not remember a visit of royalty to the outlying regions beyond the misnamed "Angel." The day of the fête is to be the 25th June, and the route of the procession, commencing at the "Angel," will be along High Street and Upper Street, as far as Highbury Station, and thence, turning suddenly to the left, the march will be direct to the hospital along the Holloway Road. The Caledonian Road, Camden Road, Parkurst and Seven Sisters Roads will be passed on the way.

The annual subscription list at the Leeds Infirmary is under £4,000. The contributions of the workpeople are also nearly £4,000, being less than £100 below the annual subscriptions. The congregational collections are only £1,799. At Leeds the workpeople do well, the wealthy anything but well; whilst the churches are decidedly stingy. There cannot be less than 300 places of worship, of all denominations, in Leeds, each of which, on an averrge, has a congregation of at least more than 100 people. If that surmise be correct, each congregation considers its duty done to the general hospital by an annual contribution of less than £6, which to each person does not represent more than a shilling per annum. Hospitals truly are noble institutions, but they are not even yet nobly supported by everybody.

The Macclesfield General Infirmary is fortunate in being endowed to a considerable extent, and in being fairly supported by the inhabitants of the town and its neighbourhood. Nevertheless the expenditure amounted last year to £3,148 2*s.* 5*d.*, which was £208 13*s.* 8*d.* more than the year's receipts. This is, however, accounted for by the fact that £326 9*s.* 6*d.* was expended in painting and renovating the Infirmary throughout. The Nurse-Training School continues to flourish, the nurses who have been trained at Macclesfield having proved very successful when they left the hospital to find work elsewhere. By means of the Thornycroft Convalescent Fund, nearly 100 patients have been sent to convalescent homes, and other convalesceut patients have received good, well-served dinners for some time after they left the Infirmary. Altogether Macclesfield seems to be doing well.

The annual gathering of the nurses of the Workhouse Infirmary Nursing Association was held on May 17th, at the house of Lady Wantage, 2, Carlton Gardens. Among the members of committee present were Mrs. Bonham Carter, the Hon. Mrs. J. G. Talbot, Miss L. Twining, Lady Colebrooke, Miss C. J. Wood, and the Hon. Secretary (Miss Wilson). Sixty nurses were present, eight being from Hampstead, and nine from St. George's-in-the-East Infirmaries; of these thirty received gratuities, and ten medals. Several of the gratuities were given after seven years' service, and the large proportion after three years' service. Miss C. J. Wood spoke to the nurses of the need of thorough training, and Miss L. Twining addressed them on the value of the National Pension Fund as a means of help in the endeavour to lay by for sickness and old age. As a guardian of the poor her experience pointed to the great need of some provision. She pointed out that from its formation the Association had incorporated in its rules a hope that each nurse should, if possible, save something during her working days. In infirmaries there was not, as in some hospitals, any charge for washing, everything being supplied in these institutions. Even from small salaries Miss Twining urged nurses to invest some amount in the fund formed for their benefit.

